# Optimizing salinity and stocking density for red tilapia in zero-water-exchange biofloc system: integrated performance, physiological, and economic assessment

**DOI:** 10.1038/s41598-025-28812-x

**Published:** 2025-12-18

**Authors:** Ghada R. Sallam, Mohamed Hamdy, Mohammed F. El Basuini, Samy Y. El-Zaeem, Yusuf Jibril Habib, Walied M. Fayed, Eslam Tefal, Akram Ismael Shehata

**Affiliations:** 1 National Institute of Oceanography and Fisheries (NIOF), Cairo City, Egypt; 2 Department of Animal and Fish Production, Faculty of Agriculture (Saba Basha), Alexandria University, Alexandria City, 21531 Egypt; 3 Department of Animal Production, Faculty of Agriculture, Tanta University, Tanta City, 31527 Egypt; 4 Faculty of Desert Agriculture, King Salman International University, Sinai City, South Sinai Egypt; 5 Department of Medical Analysis, Faculty of Applied Science, Tishk International University, Erbil City, Iraq; 6 Department of Animal and Poultry Production, Faculty of Agriculture, Damanhour University, Damanhour City, 22516 Egypt

**Keywords:** Biofloc technology (BFT), Red tilapia (*Oreochromis* spp.), Salinity, Stocking density, Growth performance, Hematological biomarkers, Antioxidant enzymes, Immune response, Biochemistry, Biotechnology, Ecology, Ecology, Ocean sciences, Physiology, Zoology

## Abstract

This study investigated the interactive effects of salinity levels (0‰, 18‰, and 36‰) and stocking densities (50, 100, 150, and 200 fish/m^3^) on water quality, growth performance, physiological responses, and economic returns of red tilapia (*Oreochromis* spp., initial weight of 12.33 ± 2.51 g/fish) reared in a biofloc technology (BFT) system using saline groundwater. A 3 × 4 factorial design with 36 fiberglass tanks (1 m^3^ each) was employed for 6 months. Key water quality indicators, fish growth indices, hematological and biochemical markers, antioxidant enzymes, immune parameters, and economic performance metrics were assessed. Results showed that increasing salinity and density significantly reduced dissolved oxygen (DO) levels and increased total ammonia nitrogen (TAN), NH_3_, NO_2_, and NO_3_ concentrations (*p* < 0.001). Biofloc volume (BFV) increased with stocking density across salinities, peaking at 44.4 ± 1.06 mL/L at 0‰ and 200 fish/m^3^, while higher salinity (36‰) generally reduced BFV. Variations in biofloc composition (protein 22–33%) and fish muscle composition (protein and lipid reduction at 36‰ and 200 fish/m^3^) indicated metabolic adjustments under stress. The highest final weight (261 ± 1.69 g/fish) was observed at 36‰ salinity with low stocking density (50 fish/m^3^), whereas the most favorable combination of growth rate, feed conversion ratio, and protein efficiency ratio occurred at 18‰ salinity and moderate stocking densities (100–150 fish/m^3^). Growth performance and feed utilization declined markedly at 36‰ with high density (200 fish/m^3^). Hematological indicators (RBC, Hb, Hct) and immune biomarkers (lysozyme, IgM, complement C3) were suppressed at extreme salinity-density combinations, while oxidative stress (high MDA) and hepatic dysfunction (elevated AST and ALT) were evident. Economic analysis confirmed that 18‰ salinity with 200 fish/m^3^ yielded the highest profit (1000 ± 54.8 EGP/treatment) and lowest operating ratio, while 150 fish/m^3^ at the same salinity provided slightly lower profit but better fish welfare indicators and immune responses, whereas high-density and hypersaline conditions reduced profitability due to poor growth and increased feed costs. In conclusion, 18‰ salinity combined with 100–150 fish/m^3^ provides the optimal balance between biological performance, fish welfare, and economic viability in red tilapia BFT systems. These findings offer evidence-based guidelines for sustainable inland saline aquaculture, supporting enhanced production efficiency and profitability in arid and saline-prone regions.

## Introduction

Aquaculture has become the fastest-growing food production sector worldwide, playing a critical role in global food security and the supply of high-quality animal protein^1,2^. As capture fisheries have reached their sustainable limits, aquaculture must expand sustainably to meet the increasing global demand for fish protein without depleting natural resources. Among modern aquaculture production systems, Biofloc Technology (BFT) has gained significant attention as an advanced and sustainable approach that improves water quality, enhances fish and shrimp growth, reduces feed costs through nutrient recycling, increases yield, minimizes water consumption, and mitigates environmental impacts^3–5^. BFT is based on the stimulation of heterotrophic and autotrophic microbial communities that aggregate into bioflocs, which simultaneously assimilate nitrogenous wastes (mainly ammonia and nitrite), improve water quality, and provide an additional protein-rich natural food source for fish. This dual role allows farmers to reduce feed costs while maintaining better environmental conditions compared to conventional systems^6^.

Despite these benefits, the success of BFT systems is strongly influenced by environmental and management factors that interact in complex ways. Previous studies have indicated that Nile tilapia (*Oreochromis niloticus*), the primary aquaculture species in Egypt, may require longer grow-out periods under BFT compared to conventional pond systems, potentially reducing profitability^7–9^. This highlights the importance of optimizing culture conditions that preserve water quality and fish health while maximizing productivity.

Freshwater scarcity is becoming an increasingly critical issue in aquaculture, particularly in arid and semi-arid regions. Factors such as climate change, groundwater depletion, and the salinization of agricultural lands reduce the availability and quality of freshwater, thereby making inland saline groundwater (SGW) an important alternative resource for aquaculture^10–12^. Salinization of agricultural lands often results from poor irrigation management and insufficient drainage^13^, excessive use of chemical fertilizers, seawater intrusion into coastal aquifers^14^, and high evaporation rates in arid and semi-arid regions. However, salinity imposes significant physiological stress on fish, as osmoregulatory mechanisms require substantial energy expenditure to maintain ion balance and osmotic homeostasis^15,16^. This redirection of metabolic energy from growth and immunity toward osmoregulation can lead to reduced feed efficiency, slower growth, impaired reproductive capacity (e.g., fecundity and offspring quality), and even survival. Developing strategies that combine salt-tolerant species with saline-adapted BFT systems offers a promising approach to sustainable aquaculture in water-limited regions.

Red tilapia (*Oreochromis* spp.) is particularly suitable for such systems due to its fast growth, efficient feed utilization, attractive coloration, high survival rates, and strong tolerance to salinity^8,17^. When integrated with BFT, red tilapia farming can significantly enhance resource efficiency. However, saline BFT systems present specific challenges, including the accumulation of suspended solids, excessive biofloc biomass, and nitrate concentrations exceeding 250 mg/L, which may result in deteriorated water quality, reduced dissolved oxygen (DO) levels, and increased biological oxygen demand (BOD), ultimately leading to physiological stress in fish^7,18–20^. Optimizing salinity levels within BFT is therefore critical to achieving stable system performance and maintaining fish welfare.

Another major factor influencing the success of intensive aquaculture is stocking density, which directly affects fish growth, feed conversion ratio (FCR), behavior, water quality, and immune responses^21,22^. While moderate increases in stocking density can enhance total yield and economic efficiency by up to 30%^23,24^, excessive crowding leads to chronic stress, which manifests as elevated cortisol levels, oxidative damage, mitochondrial dysfunction, impaired immunity, and reduced disease resistance^25,26^. These negative impacts have been observed across multiple species, including rainbow trout (*Oncorhynchus mykiss*)^27^, European seabass (*Dicentrarchus labrax*)^28^, and sturgeon (*Acipenser* spp.)^26,29^. Therefore, defining optimal stocking densities for BFT systems is essential to balance biological performance with economic profitability.

Although the individual effects of salinity and stocking density have been extensively studied, their combined impact within BFT systems remains largely unexplored^7,24,30^. In BFT, where microbial activity drives nutrient recycling and biofloc formation, salinity stress increases osmoregulatory demands, while high stocking density compounds stress by reducing dissolved oxygen and accelerating the accumulation of waste and biofloc. These interactive pressures may lead to impaired growth, reduced feed efficiency, suppressed immune responses, and ultimately compromised profitability^6,31–33^. To date, no comprehensive study has simultaneously assessed the interactive effects of these two critical factors on red tilapia reared in a zero-water-exchange saline BFT system.

This study addresses this knowledge gap by conducting a comprehensive factorial experiment to investigate the interactive effects of salinity and stocking density on the performance of red tilapia (*Oreochromis* spp.) cultured in a saline groundwater BFT system. The experiment systematically evaluated water quality dynamics, including dissolved oxygen, nitrogenous waste (ammonia, nitrite, and nitrate) accumulation resulting from feed metabolism and microbial activity, as well as biofloc composition parameters. Alongside this, we assessed growth performance, survival, and feed utilization, as well as physiological health indicators (hematological and biochemical markers), antioxidant defense mechanisms, and immune responses, in addition to the economic profitability of different culture conditions. To the best of our knowledge, this is the first study to integrate biological performance, fish welfare biomarkers, and detailed economic analysis under combined salinity-density scenarios for red tilapia in a zero-water-exchange BFT system. The findings are expected to provide evidence-based guidelines for optimizing inland saline aquaculture, enhancing both biological efficiency and economic viability in sustainable fish farming.

## Materials and methods

### Statement of ethical approval

All experimental procedures were reviewed and approved by the Animal Use Ethics Committee of Alexandria University (protocol number AU:19/24/06/11/1/34). The study was conducted following the ARRIVE guidelines v2.0 ^34^, and all methods were performed in accordance with the relevant guidelines and regulations for the ethical care and use of animals in research. Fish were handled carefully to minimize stress during all experimental procedures, and no unnecessary harm was inflicted.

### Setting up and designing the experiment

**Fig. 1:**
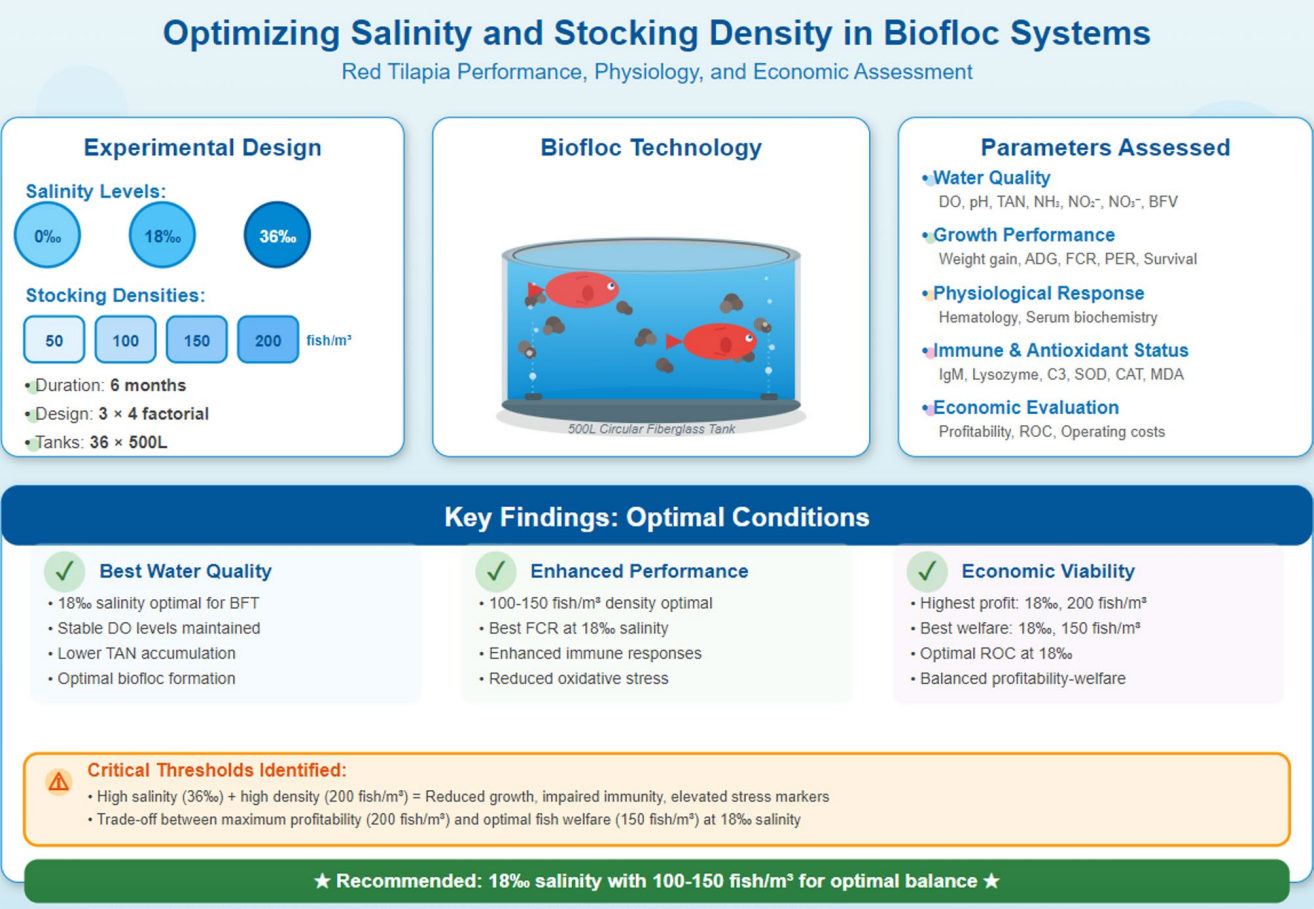
Experimental design and key findings of red tilapia (*Oreochromis* spp.) cultured in a zero-water-exchange biofloc technology (BFT) system under varying salinity and stocking density conditions.

The experiment was conducted outdoors at the El-Max Applied Research Station, Alexandria, Egypt, which operates under the National Institute of Oceanography and Fisheries (NIOF). The experimental system consisted of 36 circular fiberglass tanks, each with a capacity of 1 m^3^. To ensure durability and prevent structural deformation under water pressure, all tanks were reinforced with galvanized iron sheets. A 3 × 4 factorial design was used to evaluate the interactive effects of three salinity levels (0‰, 18‰, and 36‰) and four stocking densities (50, 100, 150, and 200 fish/m^3^) on the performance of red tilapia. Each salinity level was represented by 12 tanks, subdivided into four groups corresponding to the four stocking densities. Each treatment combination was performed in triplicate (3 tanks per treatment), resulting in a total of 12 treatment combinations and 36 experimental units. The treatments were coded as shown in Table 1. This design enabled a systematic evaluation of both the independent and interactive effects of salinity and stocking density on water quality, fish growth, physiological responses, and system performance (Fig. 1).

**Table 1:** Experimental design: salinity and stocking density treatments for red tilapia in biofloc system.

Salinity levels (‰)	Density levels (fish/m^3^)			
	D1 = 50 fish/m^3^	D2 = 100 fish/m^3^	D3 = 150 fish/m^3^	D4 = 200 fish/m^3^
S1 = 0‰ (Freshwater)	S1D1	S1D2	S1D3	S1D4
S2 = 18‰ (Brackish water)	S2D1	S2D2	S2D3	S2D4
S3 = 36‰ (Saline water)	S3D1	S3D2	S3D3	S3D4

S, salinity; D, density.

### Acclimatization of experimental fish

In early spring 2024, a total of 3000 mixed-sex red tilapia (*Oreochromis* spp.) fingerlings, with an average initial weight of 12.33 ± 2.51 g per fish, were obtained from the Lakes and Fish Resources Protection and Development Agency (LFRPDA; formerly the General Authority for Fish Resources Development, GAFRD), Ministry of Agriculture, Egypt, located at Kilometer 21 west of Alexandria. The fingerlings were originally reared in seawater at a salinity of 36‰. Upon arrival at the El-Max Applied Research Station, the fish were evenly distributed into six circular concrete tanks, each with a capacity of 5 m^3^, to initiate the acclimatization process to the target experimental salinities. The stocking density during acclimation was maintained at 100 fingerlings per cubic meter to minimize crowding stress^35,36^.

During the 24-day acclimatization period, the fish were fed twice daily with a commercial tilapia feed containing 30% crude protein (Aquafeed International for Food Industries, Motobes Industrial Zone, Kafr El-Sheikh, Egypt) (Table 2). To adjust the salinity, a mixture of fresh and saline groundwater sourced from artesian wells was used. Salinity was reduced at a constant rate of ~ 2‰ per day by partial daily replacement with freshwater, and salinity was measured daily using a calibrated refractometer to ensure accuracy. Four of the six tanks were gradually reduced to 18‰ over 10 days, while two tanks continued this reduction to 0‰ after 18 days. The remaining two tanks were maintained at the original salinity of 36‰ as the high-salinity treatment. After salinity adjustment, an additional week was allowed to ensure complete physiological adaptation of the fish to their respective salinity levels before stocking into the biofloc tanks.

**Table 2:** Formulation and proximate composition of the experimental diets (dry matter basis, DM%).

Ingredients	Amount (g/kg)	Proximate composition (%)
Fish meal (60%)	65	DM = 90.73
Shrimp meal (58%)	25	CP = 30.28
Meat and bone meal (55%)	15	EE = 6.41
Corn gluten meal (60%)	50	Ash = 8.00
Soybean meal (46%)	325	CF = 6.80
Sesame seed meal (46%)	65	NFE = 48.51
Wheat middlings (14%)	230	
Rice polishing (12%)	135	
Corn meal (8%)	60	
Vegetable oil	13	
Dicalcium phosphate	6	
Fish vitamin & mineral premix^a^	3	
DL-Methionine	1.25	
L-Lysine	1	
Choline chloride (50%)	5	
Vitamin C (Stay-C)	0.25	
Antioxidant (BHT)	0.5	
Total	1000	

Nitrogen-free extract (NFE) was calculated as: NFE = 100 – (crude protein + crude lipid + ash + crude fiber).

Ingredients were sourced from Aquafeed International for Food Industries, Motobes Industrial Zone, Kafr El-Sheikh, Egypt.

DM, dry matter; CP, crude protein; EE, ether extract (crude lipid); Ash, total mineral content; CF, crude fiber.

^a^Premix provided vitamins and minerals at recommended levels for tilapia diets.

Throughout the acclimatization phase, water quality was carefully monitored and maintained. Dissolved oxygen levels were maintained at 6.11 mg/L using continuous aeration, pH was kept within the range of 7.22–8.33, and the temperature was maintained at 26 ± 2 °C. A daily water exchange rate of 30% was conducted during acclimatization using a mixture of fresh and saline groundwater sourced from local artesian wells^37^. The tanks were illuminated by natural daylight and inspected daily to monitor fish behavior and mortality.

### Tank preparation and biofloc development

Prior to the start of the experiment, all 36 experimental fiberglass tanks were thoroughly cleaned, disinfected, and dried. All tanks were disinfected with chlorine solution (100 mg/L), rinsed three times with alternating freshwater and saline water, and thoroughly dried before use. No fish were present during the chlorination process, which followed FAO biosecurity guidelines^38,39^. After the cleaning process, biofloc development was initiated in each tank following the protocol of Sallam et al.^30^.

A carbon-to-nitrogen (C:N) ratio of 15:1 was maintained throughout the biofloc formation period using molasses as a carbon source to stimulate heterotrophic bacterial growth and promote optimal biofloc formation. The resulting nitrogen residuals, including total ammonia nitrogen (TAN), nitrite (NO_2_−), and nitrate (NO_3_−), were regularly monitored as part of the water-quality assessment (Table 3). The biofloc culture was allowed to develop and stabilize over a period of approximately 12 days prior to fish stocking. Successful biofloc formation was confirmed when a uniform brownish coloration appeared in the water, microbial aggregates were clearly visible in suspension, and the biofloc volume (BFV) measured using an Imhoff cone reached approximately 20–25 mL/L. During this period, the water in the tanks were gradually adjusted to the desired salinity levels for the experimental treatments: one-third of the tanks were adjusted to 18‰, another third to 36‰, while the remaining third were maintained as freshwater (0‰).

**Table 3:** Water quality parameters under different salinity and stocking densities in biofloc systems.

Variable	0‰				18‰				36‰				*P*-value		
	50 fish/m^3^	100 fish/m^3^	150 fish/m^3^	200 fish/m^3^	50 fish/m^3^	100 fish/m^3^	150 fish/m^3^	200 fish/m^3^	50 fish/m^3^	100 fish/m^3^	150 fish/m^3^	200 fish/m^3^	Salinity	Density	S*D
O^2^ (mg/L)	7.39 ± 0.03^a^	7.06 ± 0.04^abc^	6.7 ± 0.09^de^	6.28 ± 0.05^f^	7.29 ± 0.08^ab^	7.07 ± 0.10^abc^	7.05 ± 0.08^bc^	6.32 ± 0.06^f^	7 ± 0.06^bd^	6.85 ± 0.06^cd^	6.44 ± 0.04^ef^	6.12 ± 0.06^f^	< 0.001	< 0.001	0.021
pH	7.57 ± 0.16	7.75 ± 0.02	7.84 ± 0.03	7.6 ± 0.03	7.57 ± 0.16	7.75 ± 0.02	7.84 ± 0.03	7.6 ± 0.03	7.69 ± 0.19	7.91 ± 0.03	7.95 ± 0.03	7.51 ± 0.05	0.423	0.051	0.772
Alkalinity (as CaCO_3_) (mg/L)	135 ± 2.08^ce^	129 ± 0.58^ef^	129 ± 1.45^ef^	126 ± 0.58^f^	141 ± 0.87^acd^	137 ± 2.02^bce^	136 ± 0.44^ce^	133 ± 0.43^def^	147 ± 2.31^a^	145 ± 3.46^ab^	142 ± 2.08^ac^	137 ± 1.15^bce^	< 0.001	< 0.001	0.843
TAN (mg/L)	0.71 ± 0.01^fg^	1.00 ± 0.01^e^	1.2 ± 0.03^cd^	1.35 ± 0.03^ab^	0.47 ± 0.01^h^	0.65 ± 0.01^g^	1.1 ± 0.01^de^	1.28 ± 0.03^bc^	0.83 ± 0.02^f^	1.17 ± 0.00^cd^	1.25 ± 0.04^bc^	1.44 ± 0.06^a^	< 0.001	< 0.001	< 0.001
NH_3_ (mg/L)	0.03 ± 0.00^ef^	0.04 ± 0.01^ce^	0.05 ± 0.00^ac^	0.06 ± 0.00^ab^	0.01 ± 0.05^f^	0.02 ± 0.00^ef^	0.05 ± 0.00^bcd^	0.05 ± 0.00^ac^	0.04 ± 0.00^de^	0.05 ± 0.01^bcd^	0.06 ± 0.00^ab^	0.06 ± 0.00^a^	< 0.001	< 0.001	0.342
NO_2_ (mg/L)	0.06 ± 0.01^e^	0.08 ± 0.00^ce^	0.10 ± 0.00^acd^	0.11 ± 0.00^ab^	0.02 ± 0.00^f^	0.06 ± 0.01^e^	0.09 ± 0.00^bcd^	0.10 ± 0.00^ac^	0.08 ± 0.01^de^	0.09 ± 0.00^bcd^	0.10 ± 0.00^ac^	0.12 ± 0.01^a^	< 0.001	< 0.001	0.028
NO_3_ (mg/L)	0.31 ± 0.01^b^	0.29 ± 0.00^bd^	0.26 ± 0.01^d^	0.14 ± 0.00^e^	0.37 ± 0.01^a^	0.3 ± 0.00^bc^	0.27 ± 0.01^cd^	0.14 ± 0.01^e^	0.28 ± 0.01^bd^	0.28 ± 0.01^bd^	0.26 ± 0.01^d^	0.10 ± 0.01^f^	< 0.001	< 0.001	< 0.001
TDS (g/L)	15.7 ± 0.47^g^	29.1 ± 1.25^ef^	44.4 ± 0.63^bc^	47 ± 1.19^ab^	15.2 ± 0.3^g^	24.8 ± 0.77^f^	36.9 ± 0.52^d^	40.6 ± 2.2^cd^	15.9 ± 0.61^g^	31.3 ± 1.48^e^	48.1 ± 0.77^ab^	50.8 ± 0.98^a^	< 0.001	< 0.001	0.001
TSS (mg/L)	411 ± 7.57^bc^	401 ± 9.91^bce^	406 ± 9.48^bcd^	442 ± 5.17^ab^	356 ± 13.4^e^	360 ± 13.3^de^	376 ± 1.45^ce^	414 ± 9.96^bc^	438 ± 10.2^ab^	422 ± 10.1^ac^	422 ± 14^ac^	468 ± 5.21^a^	< 0.001	< 0.001	0.63
BFV (mg/L)	36.1 ± 0.40^bc^	39.7 ± 0.51^ab^	43 ± 0.51^a^	44.4 ± 1.06^a^	27.3 ± 0.88^ef^	32 ± 0.58^ce^	36 ± 0.58^bc^	34.3 ± 3.18^bcd^	24 ± 0.58^f^	27 ± 0.58^ef^	30 ± 0.58^de^	34 ± 0.58^cd^	< 0.001	< 0.001	0.31
Salinity (‰)	1.62 ± 0.01^e^	1.64 ± 0.01^e^	1.75 ± 0.01^e^	1.85 ± 0.03^e^	18 ± 0^d^	18.3 ± 0.16^d^	18.7 ± 0.03^c^	18.9 ± 0.07^c^	36 ± 0^b^	36.2 ± 0.09^b^	36.7 ± 0.06^a^	37 ± 0.04^a^	< 0.001	< 0.001	< 0.001

Means represent triplicate tanks per treatment (*n* = 3). Treatments followed a 3 × 4 factorial design with three salinity levels (0‰, 18‰, 36‰) and four stocking densities (50, 100, 150, and 200 fish/m^3^). Different superscript letters within rows indicate significant differences (*p* < 0.05) using two-way ANOVA with Tukey’s test.

DO, dissolved oxygen; TAN, total ammonia nitrogen; TDS, total dissolved solids; TSS, total suspended solids; BFC, biofloc volume; S×D, salinity × density interaction.

The combined biofloc formation and salinity adjustment process lasted approximately three weeks before the tanks were considered ready for fish stocking. To maintain continuous aeration and ensure the suspension of biofloc particles, each tank was supplied with a one-horsepower air blower, residues while a gasoline-powered generator was installed as a backup power source to prevent aeration failures during power interruptions. This setup ensured the stability of the biofloc system and prevented the deterioration of water quality due to oxygen depletion.

### Fish distribution and experimental setup

Following the successful stabilization of biofloc and acclimatization of the fish to the target salinity levels, a total of 2250 mixed-sex red tilapia fingerlings were randomly stocked into the 36 experimental tanks according to the assigned salinity and stocking density treatments. Mixed-sex stocks were used to better represent practical farming conditions, where mono-sex culture is not always feasible. This approach was intended to ensure broad applicability of the results rather than focusing solely on maximum growth. The experiment was conducted over six months. The fish were fed three times daily (09:00, 14:00, and 16:00 h) at a rate of 3–5% of body weight per day using a commercial floating tilapia diet containing 30% crude protein (Table 2). The feeding rate was adjusted biweekly based on biomass estimation (average weight of 60 fish per treatment, anesthetized with MS-222). This followed NRC guidelines and standard tilapia feeding tables^40^. Feed was provided in amounts adjusted biweekly based on fish biomass to maintain an optimal feeding rate while minimizing waste accumulation in the biofloc system.

### Water quality assessment

Water quality parameters were closely monitored throughout the experimental period to ensure stable environmental conditions for the fish and to evaluate the performance of the biofloc system. Daily measurements were taken for temperature, pH, and dissolved oxygen (DO), while total alkalinity, total ammonia (NH_4_^+^), unionized ammonia (NH_3_), nitrite (NO_2_−), nitrate (NO_3_−), total suspended solids (TSS), and biofloc volume (BFV) were analyzed every 10 days.

Water temperature and pH were recorded using portable pH and temperature testers (Hanna Instruments, Italy). Dissolved oxygen was measured with an EcoSense DO200A dissolved oxygen sensor (YSI Inc., USA), which was calibrated daily according to the manufacturer’s instructions. Total alkalinity, NH_3_, NO_2_−, and NO_3_− concentrations were determined spectrophotometrically using a YSI 9300 photometer. Unionized ammonia-nitrogen (NH_3_) levels were calculated following the method of Emerson et al.^41^, which incorporates pre-estimated NH_4_^+^, temperature, and pH values from the same tank. To determine TSS, 100 mL of water was collected from each tank and filtered under vacuum through pre-weighed filter paper. The filter papers were dried and weighed again to calculate the mass of suspended solids. Biofloc volume (BFV) was quantified by settling 1 L of water from each biofloc tank in an Imhoff cone for 30 min to measure the volume of settled solids, following the procedure described by Ahmad et al.^42^.

### Growth performance

The growth performance of the experimental fish was assessed biweekly to determine the effects of salinity and stocking density on key production parameters. Primary growth indicators included average initial body weight (g/fish), average final body weight (g/fish), weight gain (g/fish), average daily gain (ADG; g/fish/day), specific growth rate (SGR; %/day), survival rate (%), feed intake (FI; g/fish), feed conversion ratio (FCR), and protein efficiency ratio (PER). During each biweekly evaluation, 20 fish were randomly sampled from each replicate tank (60 fish per treatment). Fish were anesthetized with MS-222 (100 mg/L), weighed individually, and returned to tanks. Handling was minimized to reduce stress. After the 6-month trial, growth performance and feed utilization parameters were subsequently calculated in accordance with the methodologies and equations described in previous studies^30,37,43^.

### Biological indices and hematological parameters

For the blood collection and weighing process, the fish were first anesthetized with 100 mg/L MS-222 (Sigma-Aldrich) to minimize handling stress. After sampling, euthanasia was performed by immersion in an overdose of buffered MS-222 (> 300 mg/L) following American Veterinary Medical Association (AVMA: https://www.avma.org/resources-tools/avma-policies/avma-guidelines-euthanasia-animals) Guidelines (2020) for internal organs collection. Fish remained in the solution for at least 10 min after opercular movement ceased to ensure death. All procedures were approved by the Institutional Animal Ethics Committee.

At the end of the experimental period, biological indices including the viscerosomatic index (VSI %) and hepatosomatic index (HSI %) were calculated following fish dissection, according to the method described by Tefal et al.^44^. After biometric measurements, three fish from each treatment unit were randomly selected for blood collection to assess hematological parameters, following the procedures outlined in previous studies^45,46^.

Blood samples were collected directly from the caudal vein using sterilized syringes and anticoagulant-treated tubes. The total erythrocyte count (RBCs) was determined using a Neubauer hemocytometer, and results were expressed as 10^6^ cells/mm^3^, following the method of Hendricks^47^. The total leukocyte count (WBCs) was estimated using Shaw^48^ technique, with results expressed as 10^3^ cells/mm^3^. Hemoglobin (Hb) concentration was measured according to van Kampen and Zijlstra^49^, while the hematocrit (Hct) value, representing the proportion of red blood cells in the blood, was determined following Boon and BOOMS^50^.

### Proximate composition of fish and digestive enzyme assay

At the end of the trial, 30 fish from each treatment were randomly sampled to determine whole-body composition. Moisture, crude protein, crude lipid, and ash content were analyzed in both fish samples and biofloc samples, following the standard procedures of AOAC^51^. For digestive enzyme assays, five fish per tank (15 fish per treatment) were randomly selected. The intestines were carefully excised, and the intestinal contents were collected for enzymatic analysis. The contents were homogenized with a cold 0.25 M sucrose solution in glass test tubes using a Teflon-coated tissue homogenizer, then centrifuged at 5000 × g for 30 min at 4 °C. The resulting supernatant was extracted and stored at − 20 °C, with all enzyme activity measurements conducted within 24 h of extraction, as described by Makled et al.^52^.

Enzyme activities were expressed as specific activity (U/mg protein) to normalize for differences in intestinal protein content. One unit of activity was defined as the amount of enzyme required to change absorbance by 0.01 per minute under assay conditions. The protein concentration of intestinal homogenates was determined using the Bradford method for normalization^53^.

Protease activity was measured using bovine serum albumin (BSA) as the substrate, following the method described by Walter^54^, with absorbance measured at 280 nm. Amylase activity was determined using soluble starch as the substrate, based on the Bernfeld method^55^, with the release of maltose measured at 540 nm using the dinitrosalicylic acid (DNS) reagent. Lipase activity was quantified using β-naphthyl caprylate as the substrate according to Versaw et al. ^56^, with β-naphthol release measured at 410 nm. All spectrophotometric readings were conducted using a Thermo Fisher microplate reader.

### Hemato-biochemical assays

Heparinized blood samples were analyzed to determine complete blood counts (CBC), including hemoglobin (Hb), hematocrit (Hct%), red blood cell count (RBCs), and white blood cell count (WBCs). Hemoglobin and hematocrit values were measured using commercial colorimetric kits (Bio-Diagnostic Co., Cairo, Egypt), while RBC and WBC counts were quantified following the methodology described by Witeska et al.^57^. These counts were performed using an AO Bright-Line Hemocytometer (Neubauer enhanced, Precicolor HBG, Germany) for accuracy. The serum biochemical parameters evaluated included total protein (TP), albumin (Alb), total cholesterol (T-Chol), aspartate aminotransferase (AST), alanine aminotransferase (ALT), urea, and uric acid. These were measured using commercial colorimetric diagnostic kits (Bio-diagnostic Co., Giza, Egypt) following the manufacturer’s instructions and established methodologies: TP by the Biuret method^58^, Alb by the bromocresol green method^58^, T-Chol by the enzymatic CHOD-PAP method^59^, AST and ALT by the method of Reitman and Frankel^60^, urea by the urease–Berthelot method^61^, and uric acid by the uricase method^62^. Cortisol levels (pg/mL) were determined using an ELISA kit (CUSABIO, CSBE12121Fh) following the manufacturer’s guidelines. Growth hormone (GH) concentration in serum was determined using a Fish GH ELISA Kit (CUSABIO, CSBE12121Fh). The optical density (OD) for each well was read at 450 nm within 10 min using a Thermo Fisher microplate reader. GH concentrations were calculated from a standard curve generated according to the manufacturer’s instructions.

### Antioxidant enzyme activities and non-specific immunity

Serum samples were also analyzed to assess antioxidant enzyme activities and non-specific immune responses. The activity of superoxide dismutase (SOD), catalase (CAT), glutathione peroxidase (GPx) and malondialdehyde (MDA) was determined using standard colorimetric assays, following standard protocols^63–65^, respectively. The absorbance for SOD, CAT, GPx and MDA was measured at 450 nm, 405 nm, 412 nm, and 532 nm, respectively, using a microplate spectrophotometer to ensure precise and consistent measurements. For the assessment of non-specific immune responses, all enzyme activity measurements were conducted using a microplate spectrophotometer to ensure precision and analytical reliability. Serum lysozyme activity, a key indicator of innate immunity, was quantified using a turbidimetric assay based on the method described Ellis^66^. Additionally, the concentrations of complement component 3 (C3) and immunoglobulin M (IgM) were determined following the protocols outlined in previously published studies^30,37,43^.

### Economic analysis

An economic evaluation of the biofloc-based red tilapia production system was conducted using current market prices in the Arab Republic of Egypt to calculate the initial input costs and profitability. The cost of fish fingerlings was set at EGP 1.00 per fingerling, while the price of commercial feed was EGP 17.00 per kilogram, and the cost of molasses, used as a carbon source for biofloc development, was EGP 6.00 per kilogram. The selling price of harvested fish, regardless of size, was fixed at EGP 50.00 per kilogram, based on prevailing retail market values. In this economic assessment, only variable costs such as feed, fingerlings, molasses, and energy were considered, as fixed costs (e.g., infrastructure, labor, and depreciation) were assumed to remain stable across different stocking densities. The profitability and economic feasibility of the system were analyzed using profitability indices as described by Manduca et al.^67^, including net profit, profit margin, and return on cost (ROC).

### Statistical analysis

The experimental data were analyzed using a two-way analysis of variance (ANOVA) to determine the main effects of salinity and stocking density, as well as their interactions, on the measured parameters. When significant differences (*p* < 0.05) were detected, Tukey’s post-hoc test was applied to compare treatment means. The statistical methodology followed the framework outlined by Assaad et al.^68^. All results are expressed as means ± standard error of the mean (SEM).

## Results

### Water quality

The water quality parameters showed significant effects of salinity, stocking density, and their interaction for most variables (Table 3). Dissolved oxygen (DO) decreased with increasing density, with the highest value at 7.39 ± 0.03 mg/L (0‰, 50 fish/m^3^) and the lowest at 6.12 ± 0.06 mg/L (36‰, 200 fish/m^3^). pH remained stable (7.51–7.95) with a minor density effect (*p* = 0.051). Total alkalinity (CaCO_3_) increased with salinity, peaking at 147 ± 2.31 mg/L (36‰, 50 fish/m^3^).

Nitrogenous wastes (TAN, NH_3_, NO_2_) rose with higher densities (*p* < 0.001), with TAN ranging from 0.47 ± 0.01 (mg/L) (18‰, 50 fish/m^3^) to 1.44 ± 0.06 (mg/L) (36‰, 200 fish/m^3^). Total dissolved solids (TDS) and biofloc volume (BFV) also increased with both salinity and density, with BFV ranging from 24 ± 0.58 mL/L (36‰, 50 fish/m^3^) to 44.4 ± 1.06 mL/L (0‰, 200 fish/m^3^).

### Fish and biofloc composition

The proximate composition of fish muscle and biofloc varied significantly across treatments (Table 4). Fish muscle crude protein (CP) decreased significantly with increasing stocking density in the salinity treatments, but no clear density effect was observed in the freshwater control. CP values ranged from the highest (28.7 ± 0.27%) at 0‰ and 50 fish/m^3^ to the lowest (23.5 ± 0.19%) at 36‰ and 200 fish/m^3^. Muscle lipid content was consistently higher in the freshwater treatment compared to saline treatments. Within the saline groups, lipid content generally decreased with increasing salinity, except at 18‰ and 200 fish/m^3^, where values did not differ significantly from those of the freshwater control. No consistent effect of stocking density on lipid content was evident within the salinity treatments. Muscle ash content increased under higher salinity, reaching a maximum of 29.9 ± 0.06% at 36‰ and 50 fish/m^3^. In the biofloc, dry matter (DM) and CP increased with density, peaking at 33.1 ± 0.07% CP at 0‰ and 200 fish/m^3^. Biofloc lipid content decreased progressively with salinity, indicating reduced microbial lipid synthesis or greater lipid utilization under saline conditions, while ash content increased markedly with salinity, reflecting the higher accumulation of inorganic salts and minerals. The highest biofloc ash value (25.3 ± 0.09%) was recorded at 36‰ and 50 fish/m^3^ (*p* < 0.001), confirming that elevated salinity promotes mineral deposition within the floc matrix.

**Table 4:** Proximate composition of fish muscle and biofloc across treatments.

Variable	0‰				18‰				36‰				*P*-value		
	50 fish/m^3^	100 fish/m^3^	150 fish/m^3^	200 fish/m^3^	50 fish/m^3^	100 fish/m^3^	150 fish/m^3^	200 fish/m^3^	50 fish/m^3^	100 fish/m^3^	150 fish/m^3^	200 fish/m^3^	Salinity	Density	S*D
Fish															
DM (%)	27.8 ± 0.06^acd^	27.7 ± 0.03^acd^	27.2 ± 0.35^acd^	25.9 ± 0.12^de^	28.4 ± 0.24^ac^	28.1 ± 0.14^acd^	27 ± 0.39^bcd^	26.1 ± 0.37^ce^	29.4 ± 0.24^a^	29.1 ± 0.14^ab^	26 ± 1.38^de^	24.4 ± 0.02^e^	0.69	< 0.001	0.007
CP (%)	28.7 ± 0.27^a^	28.7 ± 0.19^a^	28.5 ± 0.11^a^	28.2 ± 0.12^a^	26.5 ± 0.20^b^	26.5 ± 0.03^b^	26.3 ± 0.16^b^	25.9 ± 0.22^bc^	25.1 ± 0.1^cd^	24.4 ± 0.15^d^	24.3 ± 0.12^de^	23.5 ± 0.19^e^	< 0.001	< 0.001	0.064
Lipid (%)	3.32 ± 0.01^a^	3.28 ± 0.07^a^	3.46 ± 0.03^a^	3.57 ± 0.02^a^	2.39 ± 0.01^b^	2.41 ± 0.01^b^	2.57 ± 0.06^b^	3.28 ± 0.35^a^	2.22 ± 0.06^b^	2.14 ± 0.04^b^	2.15 ± 0.03^b^	2.25 ± 0.05^b^	< 0.001	< 0.001	0.011
Ash (%)	20 ± 0.13^d^	19.8 ± 0.01^d^	19 ± 0.46^de^	18.5 ± 0.18^e^	27.2 ± 0.12^c^	27 ± 0.07^c^	26.4 ± 0.23^c^	26.2 ± 0.17^c^	29.9 ± 0.06^a^	29.4 ± 0.31^ab^	28.5 ± 0.23^b^	28.5 ± 0.24^b^	< 0.001	< 0.001	0.68
BFT															
DM (%)	22.2 ± 0.06^g^	22.3 ± 0.09^g^	22.6 ± 0.07^fg^	23.1 ± 0.06^ef^	23.3 ± 0.13^e^	23.5 ± 0.04^e^	24.7 ± 0.04^d^	25 ± 0.09^d^	26.3 ± 0.1^c^	26.7 ± 0.18^bc^	27.2 ± 0.07^ab^	27.6 ± 0.12^a^	< 0.001	< 0.001	< 0.001
CP (%)	27.9 ± 0.17^c^	30 ± 0.55^b^	32.2 ± 0.11^a^	33.1 ± 0.07^a^	25.7 ± 0.17^e^	26.2 ± 0.14^de^	28 ± 0.04^c^	30.1 ± 0.6^b^	22.4 ± 0.06^f^	22.7 ± 0.251^f^	24.9 ± 0.1^e^	27.4 ± 0.12^cd^	< 0.001	< 0.001	0.004
Lipid (%)	5.83 ± 0.14^bc^	6.15 ± 0.03^ab^	6.32 ± 0.06^ab^	6.7 ± 0.02^a^	5.17 ± 0.136^cde^	5.15 ± 0.04^de^	5.71 ± 0.11^bd^	6.08 ± 0.07^ab^	3.26 ± 0.1^g^	3.97 ± 0.13^f^	4.1 ± 0.21^f^	4.59 ± 0.27^ef^	< 0.001	< 0.001	0.175
Ash (%)	22.2 ± 0.03^d^	21.9 ± 0.09^d^	21.3 ± 0.12^e^	20 ± 0.205^f^	24.1 ± 0.07^b^	21.8 ± 0.23^de^	20 ± 0.1^f^	17.2 ± 0.07^g^	25.3 ± 0.09^a^	24.7 ± 0.09^a^	23.1 ± 0.07^c^	21.2 ± 0.06^e^	< 0.001	< 0.001	< 0.001

Means represent triplicate tanks per treatment (*n* = 3). Treatments followed a 3 × 4 factorial design with three salinity levels (0‰, 18‰, 36‰) and four stocking densities (50, 100, 150, and 200 fish/m^3^). Different superscript letters within rows indicate significant differences (*p* < 0.05) using two-way ANOVA with Tukey’s test.

DM, dry matter; CP, crude protein; S×D, salinity × density interaction.

### Growth performance and feed utilization

Growth metrics, including final weight, weight gain, and average daily gain (ADG), were significantly influenced by stocking density (*p* < 0.001), with interaction effects between salinity and density (Table 5). The highest final weight (261 ± 1.69 g/fish) and ADG (2.09 ± 0.01 g/day) were observed at 36‰ and 50 fish/m^3^; however, this treatment did not yield the best feed conversion ratio or economic return. When integrating multiple performance indicators, including growth rate, feed efficiency (lowest FCR), protein efficiency, survival, and economic outcomes, the most favorable results occurred at 18‰ salinity with moderate stocking densities (100–150 fish/m^3^). Feed conversion ratio (FCR) increased with density, reaching 1.92 ± 0.06 at 0‰ and 200 fish/m^3^, while protein efficiency ratio (PER) was highest (4.46 ± 0.15) at 18‰, 50 fish/m^3^. Total yield (T.Y) improved with density, peaking at 46.4 ± 0.92 kg/m^3^ (18‰, 200 fish/m^3^).

**Table 5:** Growth performance, feed utilization, and somatic indices of red tilapia cultured in a 3 × 4 factorial design with three salinity levels (0‰, 18‰, 36‰) and four stocking densities (50, 100, 150, 200 fish/m^3^).

Variable	0‰				18‰				36‰				*P*-value		
	50 fish/m^3^	100 fish/m^3^	150 fish/m^3^	200 fish/m^3^	50 fish/m^3^	100 fish/m^3^	150 fish/m^3^	200 fish/m^3^	50 fish/m^3^	100 fish/m^3^	150 fish/m^3^	200 fish/m^3^	Salinity	Density	S*D
Average initial weight (g/fish)	10.5 ± 0.72	11.3 ± 0.75	10.9 ± 0.2	11 ± 0.29	10.7 ± 0.80	10.4 ± 1.3	11 ± 0.90	10.9 ± 0.83	10.4 ± 0.70	11.2 ± 0.35	10.7 ± 1.24	11 ± 0.57	0.855	0.679	0.855
Average final weight (g/fish)	251 ± 2.06^a^	248 ± 1.24^a^	244 ± 3.36^a^	235 ± 2.34^a^	252 ± 6.49^a^	250 ± 8.22^a^	255 ± 1.94^a^	246 ± 2.87^a^	261 ± 1.69^a^	258 ± 2.21^a^	254 ± 10.8^a^	195 ± 7.37^b^	0.071	< 0.001	< 0.001
Weight gain (g/fish)	240 ± 1.59^a^	236 ± 1.59^a^	233 ± 3.17^a^	224 ± 2.16^a^	241 ± 6.2^a^	239 ± 7.3^a^	244 ± 2.62^a^	235 ± 2.62^a^	250 ± 1.53^a^	247 ± 2.37^a^	243 ± 12^a^	184 ± 7.87^b^	0.068	< 0.001	< 0.001
ADG (g/fish/day)	2 ± 0.01^a^	1.97 ± 0.01^a^	1.94 ± 0.03^a^	1.87 ± 0.02^a^	2.01 ± 0.05^a^	1.99 ± 0.06^a^	2.04 ± 0.02^a^	1.96 ± 0.02^a^	2.09 ± 0.01^a^	2.05 ± 0.02^a^	2.03 ± 0.1^a^	1.54 ± 0.07^b^	0.068	< 0.001	< 0.001
SGR (%/day)	2.65 ± 0.05	2.57 ± 0.06	2.59 ± 0.01	2.56 ± 0.02	2.64 ± 0.06	2.66 ± 0.08	2.62 ± 0.08	2.6 ± 0.06	2.69 ± 0.06	2.54 ± 0.03	2.65 ± 0.13	2.4 ± 0.07	0.431	0.092	0.44
Survival (%)	93 ± 0.58^abc^	91 ± 0.58^ad^	90.7 ± 0.67^bd^	89.3 ± 0.67^cd^	95 ± 0.58^a^	94.7 ± 0.33^ab^	95 ± 0.58^a^	94.3 ± 0.88^ab^	94 ± 0.58^ab^	93.3 ± 1.67^abc^	89.7 ± 0.33^cd^	87.7 ± 1.45^d^	< 0.001	< 0.001	0.021
FI (g/fish)	327 ± 4.33^cd^	331 ± 17.7^cd^	394 ± 9.76^ab^	430 ± 10.2^a^	210 ± 12.2^f^	249 ± 14.3^ef^	293 ± 2.67^cde^	290 ± 10.7^de^	319 ± 8.5^cd^	337 ± 10.3^bd^	424 ± 14.3^a^	353 ± 19.7^bc^	< 0.001	< 0.001	0.007
FCR (g)	1.36 ± 0.03^c^	1.4 ± 0.07^c^	1.69 ± 0.03^b^	1.92 ± 0.06^a^	0.87 ± 0.03^e^	1.04 ± 0.04^de^	1.2 ± 0.01^cd^	1.23 ± 0.04^cd^	1.27 ± 0.03^c^	1.36 ± 0.03^c^	1.75 ± 0.03^ab^	1.91 ± 0.06^a^	< 0.001	< 0.001	0.003
PER (g)	2.84 ± 0.05^cd^	2.77 ± 0.13^d^	2.29 ± 0.04^e^	2.02 ± 0.07^e^	4.46 ± 0.15^a^	3.73 ± 0.13^b^	3.22 ± 0.04^c^	3.14 ± 0.09^cd^	3.04 ± 0.08^cd^	2.83 ± 0.06^cd^	2.21 ± 0.04^e^	2.02 ± 0.06^e^	< 0.001	< 0.001	0.01
HSI (%)	1.97 ± 0.01^de^	2.03 ± 0.02^d^	2.29 ± 0.03^b^	1.99 ± 0.01^de^	1.96 ± 0.01^de^	1.89 ± 0.03^e^	2.04 ± 0.01^cd^	1.99 ± 0.01^de^	1.97 ± 0.03^de^	2.16 ± 0.04^bc^	2.53 ± 0.05^a^	2.66 ± 0.00^a^	< 0.001	< 0.001	< 0.001
VSI (%)	7.05 ± 0.04^cde^	7.31 ± 0.01^bd^	7.52 ± 0.02^bc^	6.67 ± 0.08^e^	6.71 ± 0.06^e^	6.77 ± 0.00^de^	6.75 ± 0.05^de^	6.67 ± 0.08^e^	7.18 ± 0.36^cde^	7.39 ± 0.02^bc^	7.84 ± 0.02^ab^	8.29 ± 0.05^a^	< 0.001	0.003	< 0.001
K	3.06 ± 0.07^a^	2.52 ± 0.05^b^	2.36 ± 0.05^b^	2.23 ± 0.01^b^	3.18 ± 0.04^a^	3.14 ± 0.10^a^	3.16 ± 0.04^a^	3.06 ± 0.07^a^	3.11 ± 0.06^a^	3.1 ± 0.07^a^	2.22 ± 0.06^b^	1.74 ± 0.09^c^	< 0.001	< 0.001	< 0.001
T.Y (kg/m)	11.6 ± 0.09^f^	22.5 ± 0.06^e^	31.7 ± 0.66^d^	42 ± 0.11^b^	12 ± 0.38^f^	23.6 ± 0.76^e^	36.4 ± 0.11^c^	46.4 ± 0.92^a^	12.3 ± 0.11^f^	24.2 ± 0.63^e^	34.1 ± 1.47^cd^	34.2 ± 1.51^cd^	< 0.001	< 0.001	< 0.001

Values are means ± SEM (*n* = 3). Treatments followed a 3 × 4 factorial design with three salinity levels (0‰, 18‰, 36‰) and four stocking densities (50, 100, 150, and 200 fish/m^3^). Means within rows sharing different superscripts differ significantly (*p* < 0.05) using two-way ANOVA with Tukey’s test.

ADG, average daily gain; SGR, specific growth rate; FI, feed intake; FCR, feed conversion ratio; PER, protein efficiency ratio; HSI, hepatosomatic index; VSI, viscerosomatic index; K, condition factor; T.Y, total yield; S×D, salinity × density interaction.

### Hematological parameters and digestive enzymes

Table 6 shows that hematological indices were significantly reduced at high densities and salinities (*p* < 0.001). RBC counts were highest at 3.10 ± 0.05 × 10^6^/mm^3^ (18‰, 50 fish/m^3^) and lowest at 1.51 ± 0.04 × 10^6^/mm^3^ (36‰, 200 fish/m^3^). Hemoglobin (Hb) and hematocrit (Hct) followed the same trend. WBC counts and lymphocyte percentages were higher at 18‰, indicating improved immune responses under moderate salinity. Digestive enzyme activities (protease, amylase, lipase) were maximized at 18‰ and 50 fish/m^3^, with protease activity at 59.9 ± 0.28 U/mg protein, but significantly dropped under 36‰ and 200 fish/m^3^ (47.1 ± 1.14 U/mg).

**Table 6:** Hematological parameters and digestive enzyme activities of red tilapia cultured under three salinity levels (0‰, 18‰, 36‰) and four stocking densities (50, 100, 150, 200 fish/m^3^) in a 3 × 4 factorial design.

Variable	0‰				18‰				36‰				*P*-value		
	50 fish/m^3^	100 fish/m^3^	150 fish/m^3^	200 fish/m^3^	50 fish/m^3^	100 fish/m^3^	150 fish/m^3^	200 fish/m^3^	50 fish/m^3^	100 fish/m^3^	150 fish/m^3^	200 fish/m^3^	Salinity	Density	S*D
RBCs (×10^6^/mm^3^)	2.87 ± 0.1^ab^	2.19 ± 0.07^cd^	1.66 ± 0.05^ef^	1.58 ± 0.03^f^	3.1 ± 0.05^a^	2.59 ± 0.15^bc^	2.08 ± 0.04^de^	1.83 ± 0.12^df^	2.87 ± 0.1^ab^	2.19 ± 0.07^cd^	1.66 ± 0.05^ef^	1.51 ± 0.04^f^	< 0.001	< 0.001	0.863
Hb (g/dL)	6.38 ± 0.09^d^	6.51 ± 0.14^d^	5.98 ± 0.08^d^	3.87 ± 0.15^e^	9.28 ± 0.23^b^	8.8 ± 0.29^bc^	7.82 ± 0.18^c^	12.5 ± 0.45^a^	6.38 ± 0.09^d^	6.51 ± 0.14^d^	5.98 ± 0.08^d^	3.87 ± 0.15^e^	< 0.001	< 0.001	< 0.001
Hct%	19.7 ± 1.35^b^	18.3 ± 0.48^bc^	17 ± 0.41^bc^	12.1 ± 0.17^cd^	27.7 ± 2.78^a^	29 ± 2.82^a^	23.9 ± 1.95^ab^	6.42 ± 0.09^d^	19.7 ± 1.35^b^	18.3 ± 0.48^bc^	17 ± 0.41^bc^	12.1 ± 0.17^cd^	< 0.001	< 0.001	< 0.001
WBCs (×10^3^ /µL)	21.9 ± 0.29^d^	21.1 ± 0.24^d^	21 ± 0.34^d^	17.2 ± 0.71^e^	35 ± 0.64^a^	33.5 ± 0.05^a^	30.6 ± 0.34^b^	26.9 ± 0.40^c^	21.9 ± 0.29^d^	21.1 ± 0.24^d^	21 ± 0.34^d^	17.2 ± 0.71^e^	< 0.001	< 0.001	0.001
Lymphocytes (%)	58.2 ± 3.82^ab^	56.7 ± 3.6^ab^	51.1 ± 4.04^bc^	37.9 ± 2.41^c^	71.5 ± 0.58^a^	70.8 ± 1.0^a^	68.7 ± 0.48^a^	66.4 ± 0.66^a^	58.2 ± 3.82^ab^	56.7 ± 3.6^ab^	51.1 ± 4.04^bc^	37.9 ± 2.41^c^	< 0.001	< 0.001	0.114
Monocytes (%)	2.27 ± 0.05^bc^	2.18 ± 0.03^cd^	1.95 ± 0.03^d^	1.31 ± 0.03^e^	2.64 ± 0.1^a^	2.54 ± 0.11^ab^	2.32 ± 0.06^bc^	2.19 ± 0.07^cd^	2.27 ± 0.05^bc^	2.18 ± 0.03^cd^	1.95 ± 0.03^d^	1.31 ± 0.03^e^	< 0.001	< 0.001	< 0.001
Neutrophils (%)	17.1 ± 0.33^d^	16.6 ± 0.23^d^	15.8 ± 0.23^d^	13.2 ± 0.26^e^	21.7 ± 0.37^a^	21.6 ± 0.69^ab^	19.9 ± 0.37^bc^	19.6 ± 0.26^c^	17.1 ± 0.33^d^	16.6 ± 0.23^d^	15.8 ± 0.23^d^	13.2 ± 0.26^e^	< 0.001	< 0.001	0.03
Protease (U/mg protein)	54.4 ± 0.40^bc^	54.2 ± 1.27^bc^	54.3 ± 0.603^bc^	48.7 ± 0.93^de^	59.9 ± 0.277^a^	59.4 ± 1.1^a^	57.4 ± 0.851^ab^	52.5 ± 0.772^cd^	58.7 ± 0.561^a^	56.9 ± 0.721^ab^	55.9 ± 0.212^ac^	47.1 ± 1.14^e^	< 0.001	< 0.001	0.03
Amylase (U/mg protein)	31.7 ± 0.54^de^	32.8 ± 0.55^cd^	32.3 ± 0.30^de^	27 ± 0.28^f^	37.5 ± 0.54^a^	37.3 ± 0.46^a^	35.6 ± 0.54^ab^	30.4 ± 0.23^e^	36.6 ± 0.33^ab^	34.7 ± 0.11^bc^	34.6 ± 0.11^bc^	25.6 ± 0.32^f^	< 0.001	< 0.001	< 0.001
Lipase (U/mg protein)	48 ± 0.32^d^	51.4 ± 0.19^c^	49.7 ± 0.13^cd^	41 ± 0.53^e^	58.4 ± 0.27^a^	57.3 ± 1.27^ab^	55 ± 0.38^b^	50 ± 1.02^cd^	56.7 ± 0.21^ab^	57 ± 0.76^ab^	54.5 ± 0.11^b^	39.2 ± 0.55^e^	< 0.001	< 0.001	< 0.001

Values are means ± SEM (*n* = 3). Treatments followed a 3 × 4 factorial design: salinity levels (0‰, 18‰, 36‰) and stocking densities (50, 100, 150, 200 fish/m^3^). Means within rows sharing different superscripts differ significantly (*p* < 0.05) using two-way ANOVA with Tukey’s test.

RBCs, red blood cells; Hb, hemoglobin; Hct, hematocrit; WBCs, white blood cells; S×D, salinity × density interaction.

### Serum biochemical, antioxidant, and immune responses

As shown in Table 7, antioxidant enzyme activities (SOD, CAT, GPx) were highest under moderate salinity and low density (e.g., SOD = 65.3 ± 0.10 U/mL at 18‰, 50 fish/m^3^), while oxidative stress (MDA) was elevated under 0‰ and 36‰ at high density (up to 0.97 ± 0.02). Liver enzymes (AST, ALT) and stress biomarkers (urea, uric acid, cortisol) increased with salinity and density, with cortisol peaking at 252 ± 0.89 pg/mL (0‰, 200 fish/m^3^). Immune markers such as IgM, lysozyme, and complement C3 were significantly suppressed at 36‰ and 200 fish/m^3^, while they peaked at 18‰ and 50 fish/m^3^ (e.g., IgM = 119 ± 1.15 µg/mL).

**Table 7:** Serum biochemical, antioxidant, and immune responses of red tilapia cultured under three salinity levels (0‰, 18‰, 36‰) and four stocking densities (50, 100, 150, 200 fish/m^3^) in a 3 × 4 factorial design.

Variable	0‰				18‰				36‰				*P*-value		
	50 fish/m^3^	100 fish/m^3^	150 fish/m^3^	200 fish/m^3^	50 fish/m^3^	100 fish/m^3^	150 fish/m^3^	200 fish/m^3^	50 fish/m^3^	100 fish/m^3^	150 fish/m^3^	200 fish/m^3^	Salinity	Density	S*D
SOD (U/mL)	63.5 ± 0.58^ab^	61.7 ± 0.45^b^	57.8 ± 0.38^c^	52.2 ± 0.52^d^	65.3 ± 0.10^a^	65 ± 0.12^a^	65.1 ± 0.11^a^	65.1 ± 0.12^a^	63.3 ± 0.55^ab^	61.6 ± 0.5^b^	58.2 ± 0.36^c^	53.6 ± 0.29^d^	< 0.001	< 0.001	< 0.001
CAT (U/mL)	16.5 ± 0.16^ab^	15.9 ± 0.21^bc^	15.3 ± 0.07^d^	14 ± 0.16^e^	17 ± 0.04^a^	17 ± 0.04^a^	17 ± 0.04^a^	17 ± 0.04^a^	16.4 ± 0.16^ab^	15.9 ± 0.21^bc^	15.4 ± 0.06^cd^	14.4 ± 0.13^e^	< 0.001	< 0.001	< 0.001
MDA (nmol/mL)	0.57 ± 0.01^cd^	0.61 ± 0.01^c^	0.71 ± 0.02^b^	0.97 ± 0.02^a^	0.52 ± 0.00^d^	0.53 ± 0.00^d^	0.52 ± 0.00^d^	0.53 ± 0.00^d^	0.57 ± 0.01^cd^	0.61 ± 0.01^c^	0.69 ± 0.02^b^	0.93 ± 0.02^a^	< 0.001	< 0.001	< 0.001
AST (IU/L)	56.3 ± 0.72^ef^	58.3 ± 0.27^ce^	60.7 ± 0.18^c^	76.1 ± 0.39^a^	55 ± 0.95^f^	56.3 ± 0.48^ef^	55.6 ± 0.72^ef^	56 ± 0.6^ef^	57.1 ± 0.48^def^	58.1 ± 0.3^ce^	59.1 ± 0.40^cd^	72.2 ± 0.56^b^	< 0.001	< 0.001	< 0.001
ALT (IU/L)	30.1 ± 0.48^b^	31.2 ± 0.61^b^	32.3 ± 0.28^b^	45.6 ± 0.33^a^	30.4 ± 0.44^b^	30.7 ± 0.31^b^	30.6 ± 0.37^b^	30.6 ± 0.34^b^	30.4 ± 0.54^b^	31.1 ± 0.47^b^	32 ± 0.45^b^	44.3 ± 0.56^a^	< 0.001	< 0.001	< 0.001
TP (g/dl)	3.76 ± 0.02^ab^	3.73 ± 0.02^ab^	3.63 ± 0.03^b^	3.13 ± 0.01^c^	3.78 ± 0.03^a^	3.74 ± 0.04^ab^	3.76 ± 0.04^ab^	3.75 ± 0.04^ab^	3.76 ± 0.02^ab^	3.72 ± 0.02^ab^	3.68 ± 0.02^ab^	3.25 ± 0.01^c^	< 0.001	< 0.001	< 0.001
Albumin (g/dl)	0.47 ± 0.03^bc^	0.47 ± 0.03^c^	0.56 ± 0.05^ac^	0.68 ± 0.02^a^	0.46 ± 0.04^c^	0.51 ± 0.05^ac^	0.49 ± 0.05^bc^	0.5 ± 0.05^ac^	0.47 ± 0.03^bc^	0.5 ± 0.04^ac^	0.5 ± 0.03^ac^	0.66 ± 0.01^ab^	0.09	< 0.001	0.096
Globulin (g/dl)	3.29 ± 0.01^ab^	3.26 ± 0.01^abc^	3.07 ± 0.03^e^	2.45 ± 0.01^g^	3.32 ± 0.01^a^	3.23 ± 0.02^bd^	3.28 ± 0.01^abc^	3.25 ± 0.01^bc^	3.29 ± 0.01^ab^	3.22 ± 0.01^cd^	3.18 ± 0.01^d^	2.59 ± 0.01^f^	< 0.001	< 0.001	< 0.001
A/G	0.14 ± 0.01^b^	0.15 ± 0.01^b^	0.18 ± 0.02^b^	0.28 ± 0.01^a^	0.14 ± 0.01^b^	0.16 ± 0.02^b^	0.15 ± 0.01^b^	0.15 ± 0.02^b^	0.14 ± 0.01^b^	0.16 ± 0.01^b^	0.16 ± 0.01^b^	0.26 ± 0.00^a^	0.001	< 0.001	< 0.001
GH (ng/ml)	2.66 ± 0.05^bc^	2.33 ± 0.16^de^	1.92 ± 0.02^fg^	1.64 ± 0.04^g^	3.09 ± 0.01^a^	2.84 ± 0.04^ab^	2.71 ± 0.06^bc^	2.09 ± 0.03^ef^	3.06 ± 0.03^a^	2.82 ± 0.02^ab^	2.58 ± 0.02^bd^	2.52 ± 0.01^cd^	< 0.001	< 0.001	< 0.001
Lysozyme (µg/mL)	1.05 ± 0.02^ab^	0.85 ± 0.02^c^	0.76 ± 0.02^cd^	0.64 ± 0.01^e^	1.15 ± 0.02^a^	1.12 ± 0.03^ab^	1.14 ± 0.02^ab^	1.13 ± 0.02^ab^	1.04 ± 0.03^b^	0.85 ± 0.01^c^	0.79 ± 0.02^c^	0.67 ± 0.02^de^	< 0.001	< 0.001	< 0.001
Urea (mg/dl)	37.7 ± 0.20^def^	38.6 ± 0.44^ce^	40.7 ± 0.3^b^	42.6 ± 0.44^a^	36.5 ± 0.12^f^	37.2 ± 0.19^ef^	36.8 ± 0.13^f^	37 ± 0.15^f^	37.9 ± 0.25^def^	38.8 ± 0.45^cd^	39.8 ± 0.06^bc^	41 ± 0.316^b^	< 0.001	< 0.001	< 0.001
Uric acid (mg/dl)	2.35 ± 0.02^ce^	2.5 ± 0.05^c^	2.82 ± 0.01^b^	3.16 ± 0.06^a^	2.12 ± 0.03^f^	2.22 ± 0.03^def^	2.17 ± 0.03^f^	2.19 ± 0.03^ef^	2.39 ± 0.01^cd^	2.51 ± 0.04^c^	2.7 ± 0.02^b^	2.87 ± 0.03^b^	< 0.001	< 0.001	< 0.001
Cortisol (pg/mL)	200 ± 1.15^cd^	209 ± 1.74^bc^	204 ± 16.4^bcd^	252 ± 0.89^a^	174 ± 2.16^e^	183 ± 2.16^de^	199 ± 1.93^ce^	201 ± 0.86^cd^	191 ± 0.47^ce^	199 ± 0.99^ce^	229 ± 2.7^ab^	243 ± 1.45^a^	< 0.001	< 0.001	0.001
Immunoglobulin M (µg/mL)	73 ± 0.58^d^	68 ± 0.58^de^	61.7 ± 1.86^ef^	58 ± 2.08^f^	119 ± 1.15^a^	114 ± 1.33^a^	104 ± 1.76^bc^	101 ± 0.88^c^	116 ± 1.26^a^	112 ± 1.53^ab^	57 ± 1.04^f^	54.7 ± 2.6^f^	< 0.001	< 0.001	< 0.001
Complement C3 (µg/mL)	62.3 ± 0.88^c^	57.7 ± 0.88^cd^	52.3 ± 0.33^de^	48 ± 0.29^ef^	88.3 ± 1.45^a^	87.6 ± 1.45^a^	86.3 ± 0.88^ab^	82 ± 1.15^b^	87.8 ± 1.45^a^	87.3 ± 1.45^ab^	46.6 ± 0.8^f^	43.3 ± 0.88^f^	< 0.001	< 0.001	< 0.001
GPx (U/mL)	2.07 ± 0.04^bc^	1.93 ± 0.01^de^	1.86 ± 0.04^e^	1.7 ± 0.04^f^	2.25 ± 0.01^a^	2.15 ± 0.02^ab^	2.12 ± 0.04^ac^	1.7 ± 0.02^f^	2.16 ± 0.02^ab^	2.04 ± 0.01^bcd^	1.99 ± 0.03^ce^	0.71 ± 0.02^g^	< 0.001	< 0.001	< 0.001

Values are means ± SEM (*n* = 3). Treatments followed a 3 × 4 factorial design: salinity levels (0‰, 18‰, 36‰) and stocking densities (50, 100, 150, 200 fish/m^3^). Means within rows sharing different superscripts differ significantly (*p* < 0.05) using two-way ANOVA with Tukey’s test. Abbreviations: SOD, superoxide dismutase; CAT, catalase; MDA, malondialdehyde; AST, aspartate aminotransferase; ALT, alanine aminotransferase; TP, total protein; A/G, albumin/globulin ratio; GH, growth hormone; IgM, immunoglobulin M; C3, complement component 3; GPx, glutathione peroxidase; S×D, salinity × density interaction.

### Economic evaluation

The economic assessment (Table 8) showed that 18‰ salinity with moderate to high densities (150–200 fish/m^3^) yielded the best economic outcomes. The highest profit (1000 ± 54.8 EGP/treatment) and lowest operating ratio (0.42 ± 0.01) were observed at 18‰ and 200 fish/m^3^. Conversely, 36‰ and 200 fish/m^3^ showed the lowest profit (150 ± 15.8 EGP) due to poor growth and higher costs. Return on costs (ROC) and profit margin were also highest under 18‰ conditions.

**Table 8:** Economic evaluation of red tilapia cultured under three salinity levels (0‰, 18‰, 36‰) and four stocking densities (50, 100, 150, 200 fish/m^3^) in a 3 × 4 factorial design.

Variable	0‰				18‰				36‰				*P*-value		
	50 fish/m^3^	100 fish/m^3^	150 fish/m^3^	200 fish/m^3^	50 fish/m^3^	100 fish/m^3^	150 fish/m^3^	200 fish/m^3^	50 fish/m^3^	100 fish/m^3^	150 fish/m^3^	200 fish/m^3^	Salinity	Density	S*D
Fish cost (LE)	50 ± 0^d^	100 ± 0^c^	150 ± 0^b^	200 ± 0^a^	50 ± 0^d^	100 ± 0^c^	150 ± 0^b^	200 ± 0^a^	50 ± 0^d^	100 ± 0^c^	150 ± 0^b^	200 ± 0^a^	0.581	< 0.001	0.386
Feed cost (LE)	278 ± 3.68^fg^	564 ± 30.1^e^	1000 ± 24.9^c^	1460 ± 34.7^a^	178 ± 10.4^g^	423 ± 24.3^ef^	748 ± 6.8^d^	988 ± 36.2^c^	271 ± 7.22^fg^	572 ± 17.4^e^	1080 ± 36.5^bc^	1200 ± 66.9^b^	< 0.001	< 0.001	< 0.001
Molas cost (LE)	37.6 ± 0.497^fg^	76.2 ± 4.08^e^	136 ± 3.37^c^	198 ± 4.7^a^	24.1 ± 1.4^g^	57.2 ± 3.29^ef^	101 ± 0.92^d^	134 ± 4.9^c^	36.7 ± 0.977^fg^	77.4 ± 2.36^e^	146 ± 4.94^bc^	162 ± 9.05^b^	< 0.001	< 0.001	< 0.001
Total cost (TC) ^a^	365 ± 4.17^fg^	569 ± 143^ef^	1290 ± 28.3^c^	1860 ± 39.4^a^	252 ± 11.8^g^	580 ± 27.6^ef^	999 ± 7.72^d^	1320 ± 41.1^bc^	358 ± 8.2^fg^	750 ± 19.8^de^	1380 ± 41.5^bc^	1560 ± 75.9^b^	< 0.001	< 0.001	< 0.001
Gross income (GI)	582 ± 4.73^f^	1130 ± 2.8^e^	1590 ± 33^d^	2100 ± 5.71^b^	598 ± 19^f^	1180 ± 38.2^e^	1820 ± 5.54^c^	2320 ± 46.2^a^	613 ± 5.57^f^	1210 ± 31.7^e^	1710 ± 73.3^cd^	1710 ± 75.4^cd^	< 0.001	< 0.001	< 0.001
Profit (P) ^b^	217 ± 8.44^ef^	558 ± 142^cd^	298 ± 18.8^ef^	243 ± 45.2^ef^	346 ± 7.33^def^	602 ± 15.6^bc^	819 ± 2.35^ab^	1000 ± 54.8^a^	255 ± 9.63^ef^	458 ± 14^ce^	329 ± 32.1^def^	150 ± 15.8^f^	< 0.001	< 0.001	< 0.001
Operating ratio ^c^	0.63 ± 0.01^bc^	0.50 ± 0.13^cd^	0.81 ± 0.01^ab^	0.89 ± 0.02^a^	0.42 ± 0.01^d^	0.49 ± 0.01^cd^	0.55 ± 0.00^cd^	0.57 ± 0.02^cd^	0.58 ± 0.01^cd^	0.62 ± 0.00^bc^	0.81 ± 0.01^ab^	0.91 ± 0.01^a^	< 0.001	< 0.001	0.012
Return on costs ^d^	1.59 ± 0.03^ab^	2.38 ± 0.79^a^	1.23 ± 0.02^ab^	1.13 ± 0.03^b^	2.37 ± 0.04^a^	2.04 ± 0.04^ab^	1.82 ± 0.01^ab^	1.76 ± 0.06^ab^	1.71 ± 0.04^ab^	1.61 ± 0.01^ab^	1.24 ± 0.02^ab^	1.1 ± 0.01^b^	0.004	0.002	0.268
Return on sales ^e^	0.37 ± 0.01^bc^	0.5 ± 0.13^ab^	0.19 ± 0.01^cd^	0.12 ± 0.02^d^	0.58 ± 0.01^a^	0.51 ± 0.01^ab^	0.45 ± 0.00^ab^	0.43 ± 0.02^ab^	0.42 ± 0.01^ab^	0.38 ± 0.00^bc^	0.19 ± 0.01^cd^	0.09 ± 0.01^d^	< 0.001	< 0.001	0.012
Profit margin ^f^	0.6 ± 0.03^ab^	1.38 ± 0.79^a^	0.23 ± 0.02^ab^	0.13 ± 0.03^b^	1.37 ± 0.04^a^	1.04 ± 0.04^ab^	0.82 ± 0.01^ab^	0.76 ± 0.06^ab^	0.71 ± 0.04^ab^	0.61 ± 0.01^ab^	0.24 ± 0.02^ab^	0.1 ± 0.01^b^	0.004	0.002	0.268

Values are means ± SEM (*n* = 3). Treatments followed a 3 × 4 factorial design: salinity levels (0‰, 18‰, 36‰) and stocking densities (50, 100, 150, 200 fish/m^3^). Means within rows sharing different superscripts differ significantly (*p* < 0.05) using two-way ANOVA with Tukey’s test.

Formulas used:

a) Total cost (TC) = cost of fish stock + cost of feed + cost of molasses.

b) Profit (P) = Gross income (GI) – Total cost (TC).

c) Operating ratio = TC/GI.

d) Return on costs = GI/TC.

e) Return on sales = P/GI.

f) Profit margin = (P/TC) × 100.

## Discussion

This investigation is one of the few comprehensive studies examining the combined effects of salinity and stocking density within biofloc technology (BFT) systems for red tilapia (*Oreochromis* spp.) culture. By evaluating environmental parameters, fish growth and physiology, immune function, antioxidant responses, and economic returns, this study provides novel insights into the threshold limits beyond which salinity and density can compromise fish performance and system stability. The findings highlight that optimal salinity (18‰) and moderate stocking densities (100–150 fish/m^3^) offered the best overall balance when growth, feed utilization, survival, immune and antioxidant status, and economic returns were considered together. While 36‰ with low density (50 fish/m^3^) produced the heaviest individual fish, this combination had lower feed efficiency and economic profitability, highlighting that “optimal” conditions are multi-criteria rather than based solely on final weight.

The results reveal that water quality parameters were significantly influenced by salinity, stocking density, and their interaction, with dissolved oxygen (DO), total ammonia nitrogen (TAN), ammonia (NH_3_), and nitrite (NO_2_) showing the most pronounced variations. The decrease in DO at higher densities and salinities, with the lowest value recorded at 6.12 ± 0.06 mg/L (36‰, 200 fish/m^3^), is consistent with previous findings in biofloc systems (BFT) where increased microbial activity consumes more oxygen due to the oxidative breakdown of organic matter and intensified respiration by heterotrophic bacteria^69,70^. Maintaining DO levels above 5–6 mg/L is critical for fish health and microbial stability in BFT ponds^71,72^. Our data indicate that while DO remained within the acceptable range, its decline under high salinity-density conditions could signal the threshold of system carrying capacity.

The pH values (7.51–7.95) remained relatively stable across treatments, showing only a minor effect of density (*p* = 0.001). This stability is typical of BFT systems, where biofloc microbial communities regulate pH through nitrification and carbon metabolism^73^. Slight fluctuations are expected due to the balance between CO_2_ release from microbial respiration and alkalinity buffering. Total alkalinity (CaCO_3_) increased with salinity, reaching 147 ± 2.31 mg/L at 36‰ and 50 fish/m^3^. This increase can be attributed to the higher ionic content and buffering capacity in saline water, which supports microbial nitrification and enhances floc formation^74,75^. Adequate alkalinity (> 100 mg/L) is essential for the nitrifying bacteria to efficiently convert ammonia into nitrite and nitrate, thus maintaining nitrogen balance^76^.

Nitrogenous wastes (TAN, NH_3_, NO_2_) exhibited a strong positive correlation with stocking density (*p* < 0.001), with TAN peaking at 1.44 ± 0.06 ppm (36‰, 200 fish/m^3^). High fish biomass and increased feed input are primary drivers of ammonia accumulation in intensive systems^77^. In BFT environments, microbial communities assimilate nitrogen into biofloc biomass; however, beyond certain thresholds of salinity and density, the microbial load may become saturated, reducing the efficiency of TAN removal^78^. Elevated ammonia levels can induce stress and gill damage in tilapia, leading to reduced growth and feed efficiency^22,79^. Our results align with findings by Ciji and Akhtar^80^, who emphasized that maintaining low TAN (< 1 ppm) is crucial to prevent sub-lethal stress.

The increase in total dissolved solids (TDS) and biofloc volume (BFV) with salinity and density is indicative of enhanced particulate organic matter and microbial proliferation. BFV ranged from 24 ± 0.58 ml/L (36‰, 50 fish/m^3^) to 44.4 ± 1.06 mL/L (0‰, 200 fish/m^3^), indicating that stocking density was the primary driver of biofloc accumulation, while higher salinity generally reduced BFV at comparable densities. This pattern suggests that dense populations promote organic waste accumulation, which fuels heterotrophic bacterial growth^3,81,82^. Although biofloc biomass provides an additional nutrient source for fish, excessive floc density may impair water clarity, reduce light penetration, and hinder fish respiration^83^. The interaction of salinity and density revealed that biofloc performance and water quality management are optimized within a narrow range. Moderate salinity (18‰) and stocking densities (100–150 fish/m^3^) maintained DO and TAN within optimal levels, indicating a balance between microbial activity and system carrying capacity. Similar findings were reported by Kumari et al.^84^ and Abdel-Rahim et al.^85^, who observed that BFT systems operated best under intermediate salinity, where microbial nitrification was most efficient.

The proximate composition of both fish muscle and biofloc exhibited significant variation across salinity and stocking density treatments. The observed changes in muscle composition indicate that red tilapia adjust their nutrient reserves in response to combined stress from salinity and density. In most saline treatments, reduced lipid levels suggest a shift toward mobilizing energy stores to sustain osmoregulatory processes. However, the lack of a significant lipid decline at 18‰ and 200 fish/m^3^ highlights that this response is not uniform, and that compensatory mechanisms may operate under certain salinity–density combinations. This highlights the importance of considering the interactive effects of multiple stressors, as fish may employ different metabolic strategies depending on the specific culture conditions. Such variability has been noted in previous studies, where tilapia and other euryhaline species exhibited context-dependent shifts in protein and lipid metabolism under osmotic stress^43,85,86^. Ash content in fish muscle increased under high salinity (up to 29.9 ± 0.06% at 36‰, 50 fish/m^3^), reflecting ionic accumulation and mineralization processes in response to elevated salinity. Similar trends have been observed in hybrid red tilapia and other euryhaline species, where increased ionic content within tissues is linked to osmoregulatory adjustments^84,87^. This mineral enrichment, while a natural adaptation, often coincides with reduced flesh quality and muscle protein content, indicating a trade-off between ionic balance and nutrient deposition.

In the biofloc (BFT), dry matter (DM) and CP content increased with stocking density, reaching a maximum CP of 33.1 ± 0.07% at 0‰ and 200 fish/m^3^. The higher protein content in biofloc under elevated densities is likely due to increased organic matter from uneaten feed and fish excreta, which supports heterotrophic bacterial growth and microbial protein synthesis^69,81,88^. This is consistent with findings by Ekasari et al.^72^, who reported that biofloc protein content rises with nutrient loading, providing a valuable supplementary protein source for cultured fish. Lipid and ash content in biofloc fluctuated with salinity, with the highest ash content (25.3 ± 0.09%) recorded at 36‰, 50 fish/m^3^. This pattern reflects changes in microbial community composition and mineral precipitation at higher salinities. Haridas et al.^89^ noted that salinity stress alters the microbial consortium in BFT, which may affect floc composition, particularly the balance between protein, lipids, and inorganic minerals. Additionally, the elevated ash content at high salinity could be linked to the accumulation of salts and mineralized waste within the floc matrix, a trend also reported in saline biofloc studies by Liu et al.^18^. The observed decline in fish muscle protein and lipid content, paired with an increase in biofloc CP, indicates a potential shift in energy and nutrient pathways under stress. While biofloc serves as an additional nutrient source, high salinity and crowding reduce the fish’s ability to efficiently assimilate these nutrients due to impaired digestion and metabolic stress^83,90^. Thus, optimal salinity-density conditions (18‰ and 100–150 fish/m^3^) appear to maximize both fish tissue quality and biofloc nutritional value.

The growth performance of red tilapia was significantly influenced by both salinity and stocking density, with clear interaction effects. The highest final weight (261 ± 1.69 g/fish) and average daily gain (ADG; 2.09 ± 0.01 g/day) were recorded at 36‰ salinity and 50 fish/m^3^, indicating that low stocking density combined with elevated salinity can initially enhance somatic growth due to reduced competition and a lower osmoregulatory burden. This observation aligns with previous reports, which found that moderate salinity can improve growth rates in *Oreochromis* spp. by reducing the energy required for osmoregulation, thereby increasing energy allocation for growth^84,87^. However, at 36‰ and 200 fish/m^3^, both final weight (195 ± 7.37 g/fish) and ADG (1.54 ± 0.07 g/day) decreased sharply, reflecting the synergistic negative effects of osmotic stress and crowding. High-density environments increase competition for feed and oxygen, while elevated salinity intensifies energy demands for maintaining ion balance. Together, these stressors compromise feed intake, growth efficiency, and fish welfare^22,29^.

Feed conversion ratio (FCR) increased with stocking density, reaching 1.92 ± 0.06 at 0‰ and 200 fish/m^3^, suggesting reduced feed utilization efficiency under crowded conditions. This aligns with earlier studies indicating that high stocking densities increase maintenance energy requirements and reduce feeding efficiency^52,91^. In contrast, the lowest FCR values and highest protein efficiency ratio (PER; 4.46 ± 0.15) were achieved at 18‰ and 50 fish/m^3^, emphasizing that moderate salinity enhances nutrient assimilation while reducing energy loss due to osmoregulatory stress. Similar trends in improved FCR and PER under optimal salinity levels have been reported in BFT-reared *O. niloticus*^78,90^. Interestingly, total yield (T.Y) improved with density, peaking at 46.4 ± 0.92 kg/m^3^ (18‰, 200 fish/m^3^). While higher density increases biomass output, it does not necessarily correlate with individual growth performance or feed efficiency. The trade-off between total production and individual fish welfare is evident, as high-density conditions often lead to slower growth, increased stress, and poorer flesh quality^8,85^. The current findings suggest that 18‰ salinity with 100–150 fish/m^3^ offers the best compromise between high total yield, optimal feed utilization (low FCR), and individual fish growth. Beyond these thresholds, both physiological stress (e.g., elevated cortisol, oxidative stress) and economic inefficiencies (high feed costs per unit biomass) become significant, as highlighted by previous bioeconomic analyses of tilapia BFT systems^24,67^.

The hematological indices reveal a marked decline in red blood cell (RBC) count, hemoglobin (Hb), and hematocrit (Hct) at high salinity-density combinations (36‰, 200 fish/m^3^), with RBC levels dropping to 1.51 ± 0.04 × 10^6^/mm^3^, compared to 3.10 ± 0.05 × 10^6^/mm^3^ at 18‰ and 50 fish/m^3^. Such reductions indicate impaired oxygen-carrying capacity and potential anemic conditions under chronic stress, consistent with previous findings in tilapia reared under high stocking densities or salinity stress^77,85,92^. Elevated energy demands for osmoregulation at high salinity can suppress erythropoiesis and deplete hematological reserves^87^. White blood cell (WBC) counts and lymphocyte percentages were higher at 18‰ salinity, particularly at moderate densities, suggesting a more robust immune response under these conditions. Moderate salinity may enhance the activity of non-specific immune cells due to reduced physiological stress, while extreme conditions (36‰, 200 fish/m^3^) lead to immunosuppression, as reflected in lowered WBC and lymphocyte values. Similar immune suppression under combined osmotic and density stress has been reported in *O. niloticus* and other BFT species^93,94^. Digestive enzyme activities (protease, amylase, and lipase) followed a similar trend, peaking at 18‰ and 50 fish/m^3^, with protease activity reaching 59.9 ± 0.28 U/mg, but declining significantly under 36‰ and 200 fish/m^3^ (47.1 ± 1.14 U/mg). This reduction can be attributed to stress-related impairment of gastrointestinal function, as stress hormones such as cortisol are known to suppress digestive enzyme secretion and gut motility^52,91^. Conversely, optimal activity at moderate salinity reflects efficient nutrient assimilation, contributing to improved FCR and PER under these conditions. The observed hematological and digestive patterns confirm that 18‰ salinity with moderate stocking densities (100–150 fish/m^3^) creates a physiological environment that supports both immune competence and digestive efficiency, thereby optimizing overall growth and health. These results are in line with previous studies, which emphasize that well-balanced biofloc systems can enhance both immune and digestive responses due to the continuous provision of microbial protein and bioactive compounds^8,84^.

The serum biochemical and antioxidant profiles highlight the strong influence of salinity-density interactions on oxidative stress, liver function, and immune responses in red tilapia cultured in BFT systems. Antioxidant enzymes superoxide dismutase (SOD), catalase (CAT), and glutathione peroxidase (GPx) showed maximum activity at 18‰ salinity and low density (50 fish/m^3^), with SOD reaching 65.3 ± 0.10 U/mL. This suggests that moderate stress levels in BFT can stimulate antioxidant defenses, enabling the fish to neutralize reactive oxygen species (ROS) effectively^94,95^. However, at extreme conditions (36‰ salinity, 200 fish/m^3^), antioxidant enzyme activities declined sharply, while malondialdehyde (MDA), a marker of lipid peroxidation, peaked at 0.97 ± 0.02 nmol/mL, indicating oxidative damage. Similar biphasic antioxidant responses have been documented in tilapia and prawns reared under high stocking densities in biofloc systems, where prolonged exposure to stress depletes the fish’s endogenous antioxidant reserves^96,97^.

Liver enzymes (AST, ALT) were significantly elevated under high salinity and density conditions, reflecting hepatic stress and metabolic overload. This pattern aligns with findings from Shehata et al.^98^, who noted that chronic stress impairs liver function in *O. niloticus*. Additionally, urea and uric acid levels increased with salinity, suggesting enhanced protein catabolism and compromised nitrogen metabolism, particularly under osmotic stress^99^. The stress hormone cortisol reached its highest value (252 ± 0.89 pg/mL) at 0‰ salinity and 200 fish/m^3^, confirming that crowding and inappropriate salinity amplify the stress response. Elevated cortisol levels are well-known indicators of chronic stress in fish, often associated with immune suppression and reduced growth performance^27,100^. Immune markers (IgM, lysozyme, complement C3) displayed a distinct pattern, highest values were observed at 18‰, 50 fish/m^3^ (e.g., IgM = 119 ± 1.15 µg/mL), indicating optimal immune competence. Lowest values occurred at 36‰, 200 fish/m^3^, suggesting immunosuppression under combined osmotic and crowding stress. This aligns with previous studies on tilapia and catfish in biofloc environments, where bioactive components in the floc (e.g., polysaccharides, vitamins, carotenoids) enhanced immune activity at optimal culture conditions, but failed to offset severe stress at extreme conditions^19,75,93^. The combined patterns of elevated cortisol, liver enzymes, and MDA, with reduced IgM and C3, strongly suggest that 36‰ salinity and 200 fish/m^3^ surpass the physiological stress threshold for red tilapia in BFT systems. In contrast, 18‰ salinity with 100–150 fish/m^3^ not only maximized antioxidant activity and immune responses but also minimized stress biomarkers, indicating the optimal biological window for growth and health.

The economic analysis reinforces the biological findings by showing that 18‰ salinity with moderate to high stocking densities (150–200 fish/m^3^) provided the most favorable economic outcomes. The highest recorded profit (1,000 ± 54.8 EGP/treatment) and lowest operating ratio (0.42 ± 0.01) occurred at 18‰ and 200 fish/m^3^, reflecting the synergistic benefits of optimal water quality, feed utilization, and growth performance under these conditions. However, 18‰ with 150 fish/m^3^ produced a slightly lower profit but offered improved welfare-related indicators (e.g., higher immune responses, lower oxidative stress), suggesting it may be the preferred compromise between profitability and fish health. Similar bioeconomic advantages of moderate salinity have been documented in biofloc-reared tilapia and shrimp, where improved FCR directly translates into reduced feed costs and higher net returns^24,67^. In contrast, 36‰ salinity at 200 fish/m^3^ yielded the lowest profit (150 ± 15.8 EGP) due to a combination of poor growth performance (low final weight and ADG), elevated feed costs, and increased energy demands for osmoregulation. These factors contributed to a higher operating ratio (0.91) and lower return on costs (ROC), indicating that hypersaline conditions beyond the physiological tolerance of red tilapia are economically unsustainable. Such findings are consistent with observations by Rahman et al.^22^, who reported that excessive salinity leads to reduced feed efficiency and higher production costs due to prolonged rearing periods.

The profit margin and ROC were highest under 18‰ salinity across all densities, with maximum cost-effectiveness observed at 150 fish/m^3^. This density provided the optimal balance between biomass yield (total yield = 46.4 ± 0.92 kg/m^3^) and feed efficiency, thereby maximizing net returns. Increased density beyond this point (e.g., 200 fish/m^3^) did not significantly improve profitability due to diminishing growth rates and potential stress-related feed inefficiencies, as noted in intensive tilapia systems^27^. Furthermore, the economic evaluation aligns with biological indicators such as the low FCR (0.93–1.10) and high protein efficiency ratio (PER), which significantly reduce feed costs the primary expense in aquaculture operations^94^. Biofloc technology (BFT) contributes to cost reduction by providing a supplemental protein source (microbial biomass), enhancing feed utilization, and minimizing water exchange costs, which further improves profitability^8^.

Integration of water quality, growth, physiological, and economic data revealed distinct salinity-density thresholds governing biofloc system performance. Optimal biological and economic outcomes occurred at 18‰ and 150–200 fish/m^3^, where nitrogenous wastes remained within acceptable limits, antioxidant defenses were maintained, and feed conversion efficiency supported the highest profitability. However, this density induced moderate immune suppression and oxidative stress compared to lower densities, indicating a trade-off between maximum yield and fish welfare. At extreme conditions (36‰ and 200 fish/m^3^), reduced digestive enzyme activities, elevated liver enzymes (AST, ALT), and suppressed IgM and complement C3 indicated systemic physiological breakdown. Conversely, biofloc composition adapted to stress via increased crude protein, suggesting microbial resilience under adverse conditions. Economic modeling confirmed that profits declined sharply under hypersaline, high-density conditions due to feed inefficiencies and health-related performance losses. These findings emphasize the need for integrated biofloc management, where salinity and density are jointly optimized to balance production efficiency, fish health, and economic sustainability.

## Conclusion

This study shows that both salinity and stocking density have considerable influence on the biological, physiological, and economic factors of red tilapia grown in a biofloc system using saline groundwater. High salinity (36‰) and high stocking density (200 fish/m^3^) made the water quality worse, slowed growth, and caused physiological stress, as shown by worse hematological and immunological indicators, more oxidative damage, and less efficient feed use. Moderate salinity (18‰) with high density (200 fish/m^3^) generated the highest economic return (1000 ± 54.8 EGP/treatment) and lowest operating ratio, while 18‰ with medium density (150 fish/m^3^) produced slightly lower profit but yielded better welfare-related outcomes, including stronger immune responses, lower oxidative stress, and improved hematological indices. These findings highlight a trade-off between maximum profitability and optimal fish health. For producers prioritizing economic output, 18‰ and 200 fish/m^3^ may be recommended, whereas those aiming for a balanced approach that safeguards fish welfare should consider 18‰ and 150 fish/m^3^. In both cases, careful management of salinity and density is essential to sustain water quality, resource efficiency, and long-term viability of saline biofloc aquaculture systems. If these best practices were used, they might greatly boost productivity in areas where freshwater is scarce, making aquaculture more robust and profitable.

## Data Availability

Data are available from the corresponding author upon reasonable request.
